# Association between serum transferrin saturation levels and heart failure in adults aged ≥40 years: a cross-sectional study based on NHANES (2017-2020.03)

**DOI:** 10.3389/fendo.2024.1419064

**Published:** 2024-08-30

**Authors:** Mian Wang, Dongyang Zhang, Lanying Jiang, Maosheng Ye, Jing Nie, Junjie Yin

**Affiliations:** ^1^ Department of Geriatric, Zhejiang Hospital of Integrated Traditional Chinese and Western Medicine, Hangzhou, Zhejiang, China; ^2^ Department of Hepatobiliary and Pancreatic Surgery, Affiliated Hangzhou First People’s Hospital, School of Medicine, Westlake University, Hangzhou, Zhejiang, China

**Keywords:** transferrin saturation, heart failure, NHANES, weighted logistic regression analysis, subgroup analysis

## Abstract

**Background:**

Limited data are available regarding the association between serum transferrin saturation (TSAT) levels and heart failure (HF).

**Methods:**

We utilized data from National Health and Nutrition Examination Survey (NHANES) 2017- 2020.03 for analysis. Data on TAST, HF and covariates were extracted and analyzed. Weighted logistic regression and subgroup analysis were used to explore the independent association between TSAT and HF. Furthermore, interaction tests were also carried out to evaluate the strata differences. We subsequently assessed whether there was a non-linear relationship between the 2 using Restricted cubic spline (RCS) and threshold effect models.

**Result:**

A total of 282 (3.87%) participants were identified to have HF. Among the total population, participants with HF had significantly lower TSAT levels compared to those without HF (24.63 vs. 27.95, P = 0.001). After fully adjusting for potential confounders, weighted multiple logistic regression models revealed a 2.6% reduced in the risk of HF when each unit of TSAT increased. There was also a negative association between elevated TSAT and developed risk of HF in the quartile groups (Q1 OR:1.00; Q2 OR: 0.924 [95%CI:0.593,1.440]; Q3 OR: 0.515 [95%CI:0.298,0.891]; Q4 OR:0.411 [95%CI:0.201,0.839]). The subgroup analysis results remained consistent across strata, with a strong negative correlation between TSAT and HF. Interaction tests showed no dependence on gender, age, Body Mass Index, race, diabetes, hypertension, hyperlipidemia, ratio of family income to poverty and education for this negative association between TSAT and HF (all p for interaction >0.05). The RCS and threshold effect models indicated a linear negative correlation between TSAT and HF, which was more pronounced when TSAT under 40%.

**Conclusion:**

Overall, these findings suggest a consistent and negative association between TSAT levels and the presence of HF among middle-aged and older adults in the United States.

## 1 Introduction

Heart failure (HF) is the terminal stage of numerous cardiovascular diseases, leading to diminished functional capacity, poorer quality of life, higher mortality rates, thus posing a significant public health concern (1). Despite considerable advancements in HF treatment in recent years, patient mortality and re-hospitalization rates remain alarmingly high.

Iron deficiency (ID) is a common comorbidity in patients with HF and is independently linked to adverse outcomes (2–4). The European Society of Cardiology (ESC) guidelines for ID primarily focus on ferritin or transferrin saturation (TSAT) combined with ferritin (5). However, ferritin levels can be influenced by inflammatory pathways activation, especially in patients with chronic inflammatory diseases, and TSAT is less likely to fluctuate than ferritin, so TSAT is most associated with diagnostic ID (6, 7). Based on this, we speculate that TSAT has a greater impact on adverse outcomes in patients with HF. Current studies have mainly focused on adverse outcomes in patients with HF (8, 9), and the relationship between TSAT and the incidence of HF remains unknown.

Therefore, we performed a cross-sectional analysis to investigate the potential association between TSAT and HF in the United States adults.

## 2 Materials and methods

### 2.1 Data source

The National Health and Nutrition Examination Survey (NHANES) is a rigorous and scientific database, which provides a large amount of information on the nutrition and health of the non-institutionalized the United States population. Employing a stratified multistage probability sampling method, NHANES ensures the selection of a highly representative sample. Approved by the Ethic Review Board of the National Center for Health Statistics (NCHS), NHANES protocols involve both interviews and examinations, with all participants providing consent for their information to be used in research. The Institutional Review Board at the Zhejiang Hospital of Integrated Traditional Chinese and Western Medicine determined that this analysis used public datasets thus human subject approval was not needed.

### 2.2 Study population

**Figure 1:**
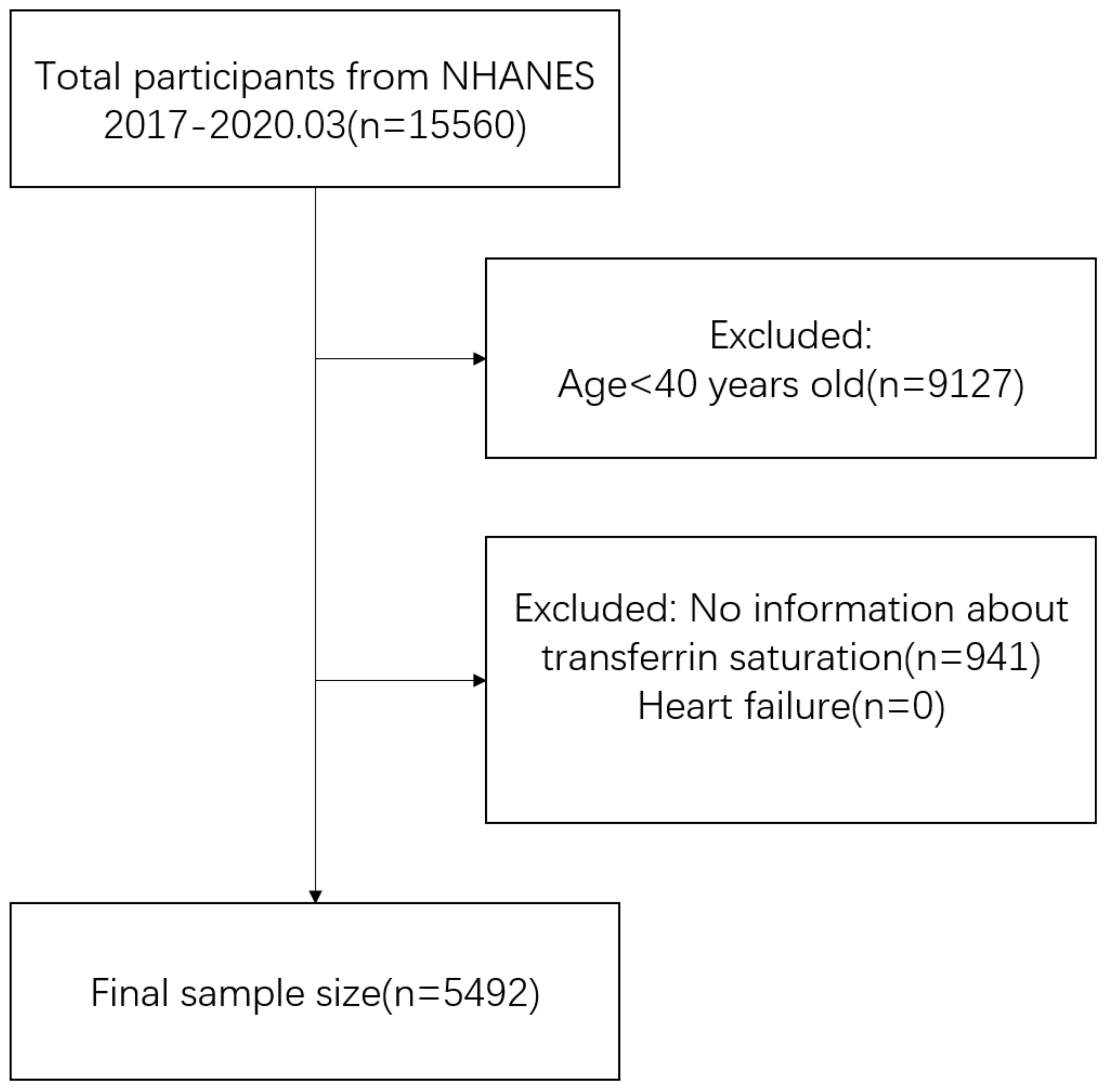
Flow chart for participants.

The study incorporated data from a single cross-sectional cycle of NHANES, which began in 2017 and ended in March 2020. Inclusion criteria comprised middle-aged and elderly (aged ≥40 years) individuals with complete TSAT and HF data (10, 11). Participant selection details are illustrated in **Figure 1**. Data on TSAT, HF and potential confounding factors were extracted and aggregated for each participant. Ultimately, the analysis included a total of 5,492 participants who met the specified inclusion and exclusion criteria.

### 2.3 Heart failure

The outcome was HF. We defined HF by a synthesis of self-reported physician diagnoses and standardized medical condition questionnaires completed during personal interviews. HF was defined as individuals who responded affirmatively to the question MCQ160b ‘Has a doctor or other health professional ever told {you/SP} that {you/s/he}…had congestive heart failure?’

### 2.4 Transferrin saturation

Serum TSAT levels was used as the primary exposure variable of interest. TSAT(%sat) value was calculated using Iron (frozen), serum and calculated Total Iron Binding Capacity (TIBC): calculated %Sat= [Iron (frozen), serum/TIBC] x 100.

### 2.5 Other covariates

Theoretically, factors with potential effects on TSAT and HF should be adjusted for as covariates in a multiple logistic regression model, but such study conditions may not be met in practice. Therefore, based on previous studies, we have used well known variables as covariates in this study (12, 13). Demographic information included age (years), Body Mass Index (BMI, <25/25-30/>30), gender (Male/Female), race (Mexican American/Non-Hispanic White/Non-Hispanic Black/Other Race), education level (Less than high school/High school/More than high school), marital status (married or living with partner/living alone) and ratio of family income to poverty (PIR, ≤1.30/1.31-3.5/>3.5). Smoking and alcohol consumption were obtained from the questionnaire. Smoking status were categorized as never, former, current), and drink status were categorized as never, sometimes, often. Medical conditions were obtained from questionnaires, including hypertension (yes/no), diabetes (yes/no) and hyperlipoidemia (yes/no). All demographic information were used as covariates in the model and adjusted to exclude the effect of disease on the independent and dependent variables. Participants’ daily intake of total nutrients (energy, iron, sodium) was obtained through a questionnaire and calculated as the average of the sum of nutrient values answered on the first and second day. Sedentary minutes were obtained from the physical activity questionnaire, where we directly collected the amount of time (mins) participants were sedentary and inactive. HF can lead to liver and kidney dysfunction (14–16), so we obtained data on aspartate aminotransferase (AST, U/L), alanine aminotransferase (ALT, U/L), albumin (g/dL), blood urea nitrogen/creatinine (BUN/Cr) from the laboratory data. In addition, HF has been associated with chronic inflammation (17, 18), so we obtained high-sensitivity C-reactive protein data (HS-CRP, mg/L) from the laboratory data. Serum iron (ug/dL) and hemoglobin (g/dL) have been clearly associated with TSAT (19, 20), so we collected data on these two. All covariates are listed in **Table 1**.

**Table 1:** Characteristics of the study population, NHANES 2017-2020.03.

Characteristics	Non-HF (n = 5210)	HF (n = 282)	P value
Demographics			
Age (year)	58.71±11.64	67.04±10.10	<.001
Age (%)			<.001
40≤Age<60	2741 (58.0)	68 (26.7)	
60≤Age ≤ 80	2469 (42.0)	214 (73.3)	
BMI (kg/m2)	29.96±6.79	33.31±8.08	<.001
BMI (%)			<.001
<25	1138 (21.9)	38 (11.0)	
25-30	1806 (37.0)	68 (24.7)	
>30	2171 (41.2)	166 (64.3)	
Gender (%)			0.004
Male	2547 (47.1)	167 (57.9)	
Female	2663 (52.9)	115 (42.1)	
Race (%)			0.003
Mexican American	579 (6.8)	15 (2.8)	
Non-Hispanic White	1914 (67.1)	136 (72.8)	
Non-Hispanic Black	1316 (9.9)	89 (14.8)	
Other Race	1401 (16.3)	42 (9.6)	
Marital status (%)			0.014
Married/Living with partner	3177 (67.4)	142 (57.7)	
Widowed/Divorced/ Separated/Never married	2033 (32.6)	140 (42.3)	
Education level (%)			0.071
Less than high school	476 (4.4)	29 (7.0)	
High school	574 (7.6)	39 (12.3)	
More than high school	4160 (88.0)	214 (80.7)	
PIR (%)			0.006
≤1.30	1144 (13.5)	77 (21.0)	
1.31-1.85	1761 (30.5)	121 (44.2)	
>1.85	1585 (44.8)	51 (27.3)	
Not recorded	720 (11.2)	33 (7.5)	
Smoking status (%)			0.001
Never	2917 (54.9)	107 (40.8)	
Former	1438 (30.5)	124 (45.5)	
Current	855 (14.6)	51 (13.7)	
Drinking status (%)			0.042
Never	1209 (19.8)	104 (29.5)	
Sometimes	1302 (30.0)	52 (24.6)	
Often	2699 (50.2)	126 (45.9)	
Hypertension (%)			<.001
Yes	2965 (50.9)	239 (82.7)	
No	2245 (49.1)	43 (17.3)	
Diabetes (%)			<.001
Yes	1395 (21.1)	151 (50.7)	
No	3815 (78.9)	131 (49.3)	
Hyperlipidemia (%)			0.526
Yes	3969 (76.8)	215 (79.3)	
No	1241 (23.2)	67 (20.7)	
Laboratory indices			
TSAT (%)	27.95±11.08	24.63±10.39	0.001
AST (U/L)	21.97±13.13	20.88±7.78	0.115
ALT (U/L)	22.34±16.14	19.44±9.29	0.007
Albumin (g/dL)	4.06±0.31	3.93±0.36	<.001
Serum Iron (ug/dL)	88.78±33.20	75.94±28.54	<.001
BUN/Cr	18.51±5.79	19.22±6.60	0.178
Hemoglobin (g/dL)	14.17±1.46	13.71±1.73	0.002
HS-CRP(mg/L)	3.95±8.17	7.81±16.00	0.024
Daily Intake			
Energy (Kcal)	2123.11±910.06	2035.60±1095.32	0.387
Iron (mg)	13.82±7.76	13.57±8.42	0.754
Sodium salt (mg)	3386.47±1698.44	3298.04±1865.02	0.686
Sedentary (mins)	401.32±698.43	475.06±895.51	0.194

### 2.6 Statistical analysis

Sample weights were incorporated into all estimates. Continuous variables were presented as mean (standard error), while categorical variables were represented as percentages.

The statistical analysis consisted of four main steps to investigate the association between TSAT and HF among the selected participants. Firstly, participants were divided into HF and non-HF groups. Weighted Student t test (for continuous variables) and weighted chi-square tests (for categorical variables) were conducted to compare the baseline characteristics. Secondly, weighted multivariable logistic regression analysis was performed to assess the relationship between TSAT and HF. Three models were utilized: Model 1 without covariate adjustments. Model 2 adjusted for gender, age and race. Model 3 adjusted all demographic information. Thirdly, a subgroup analysis was conducted to explore the impact of different subgroups on the results. Interaction tests were employed to explore potential heterogeneity between these subgroups. Additionally, Restricted cubic spline (RCS) was applied to examine linear or nonlinear relationship between them. Threshold effect analysis was conducted using two-piecewise linear regression model.

The presence of missing covariates in cross-sectional studies is inevitable, and in order to retain more information about the independent and respondent variables, missing covariates were processed. If the missing value of a continuous variable was within 10% of the total sample, we used the mean instead, otherwise we grouped the continuous variable according to certain rules, setting the missing value in a separate group. If the missing value of the categorical variable did not exceed a sample size of 20, we were directly regard as ‘no’ or ‘intermediate column’, otherwise we group them separately (21). If only the nutrient (energy, iron, sodium) of the second day is missing, the average nutrient (energy, iron, sodium) is replaced with the nutrient (energy, iron, sodium) of the first day. If the nutrients (energy, iron, sodium) are missing for both days, then proceed in the same way as the continuous variable.

Data processing and analysis were performed using R version 4.3.0, along with Storm Statistical Platform (http://www.medsta.cn/software), with P<0.05 considered to be statistically significant.

## 3 Results

### 3.1 Characteristics of participants

**Table 1** presents the weighted baseline characteristics of participants selected from NHANES 2017 to 2020.03, categorized by the presence or absence of HF. The analysis encompassed 282 participants diagnosed with HF, constituting 3.87% of the total sample. The average age of the HF group was 67.04 years, with 57.9% being male and 42.1% female. In contrast, the non-HF group comprised 5,210 participants with an average of 58.71 years, of which 47.1% were male and 52.9% were female.

Significant differences between the two groups were noted in several including TSAT, ALT, albumin, serum iron, hemoglobin, HS-CRP, age, BMI, gender, race, PIR, marital status, smoking status, drinking status, hypertension and diabetes (all p < 0.05). There were no differences in education, hyperlipidemia, AST, BUN/Cr, daily intake and sedentary time (p > 0.05).

### 3.2 Association between TSAT and HF

**Table 2** presents the association between TSAT and the risk of HF. Three multivariate logistic regression models were employed to assess this association. In Model1, TSAT exhibited a negative correlation with the risk of HF (OR=0.970, 95%CI:0.952-0.989, P<0.05). This negative association remained significant after adjusting for confounding factors in both Model2 (OR=0.962, 95%CI:0.939-0.985, P<0.05) and Model3 (OR=0.974, 95%CI:0.950-0.998, P<0.05).

**Table 2:** Weighted Multivariate logistic regression models of TSAT with HF.

Outcome	Model 1		Model 2		Model 3	
	OR (95%CI)	P value	OR (95%CI)	P value	OR (95%CI)	P value
TSAT	0.970(0.952,0.989)	0.003	0.962(0.939,0.985)	0.002	0.974(0.950,0.998)	0.034
Categories						
Q1	Reference		Reference		Reference	
Q2	0.942(0.635,1.399)	0.758	0.795 (0.526,1.201)	0.258	0.924(0.593,1.440)	0.700
Q3	0.523(0.350,0.781)	0.003	0.401(0.254,0.633)	<.001	0.515(0.298,0.891)	0.022
Q4	0.366(0.204,0.659)	0.002	0.292(0.148,0.577)	0.001	0.411(0.201,0.839)	0.020
P for trend	<.001		<.001		0.003	

Model 1: no covariates were adjusted. Model 2: Age, gender and race were adjusted. Model 3: All demographic information were adjusted. The stratification variable itself in each subgroup analysis was not included in the adjusted model. P for interaction were from the likelihood ratio test.

To delve deeper into the relationship between TSAT and HF, TSAT was categorized into quartiles. In Model3, the third quartiles (Q3: OR=0.515(0.298,0.891), 0.022) and highest (Q4: OR=0.411(0.201,0.839),0.020) showed a more pronounced negative correlation with the risk of HF compared to the lowest quartile (Q1). However, the second quartile (Q2: OR=0.924(0.593,1.440),0.700) did not exhibit statistical significance compared to the lowest quartile(Q1). These findings were consistent across all models. Additionally, when stratified by TSAT, the trend test between them remained significant in all models (P for trend<0.05).

### 3.3 Relationships of TSAT with HF in subgroups

**Figure 2:**
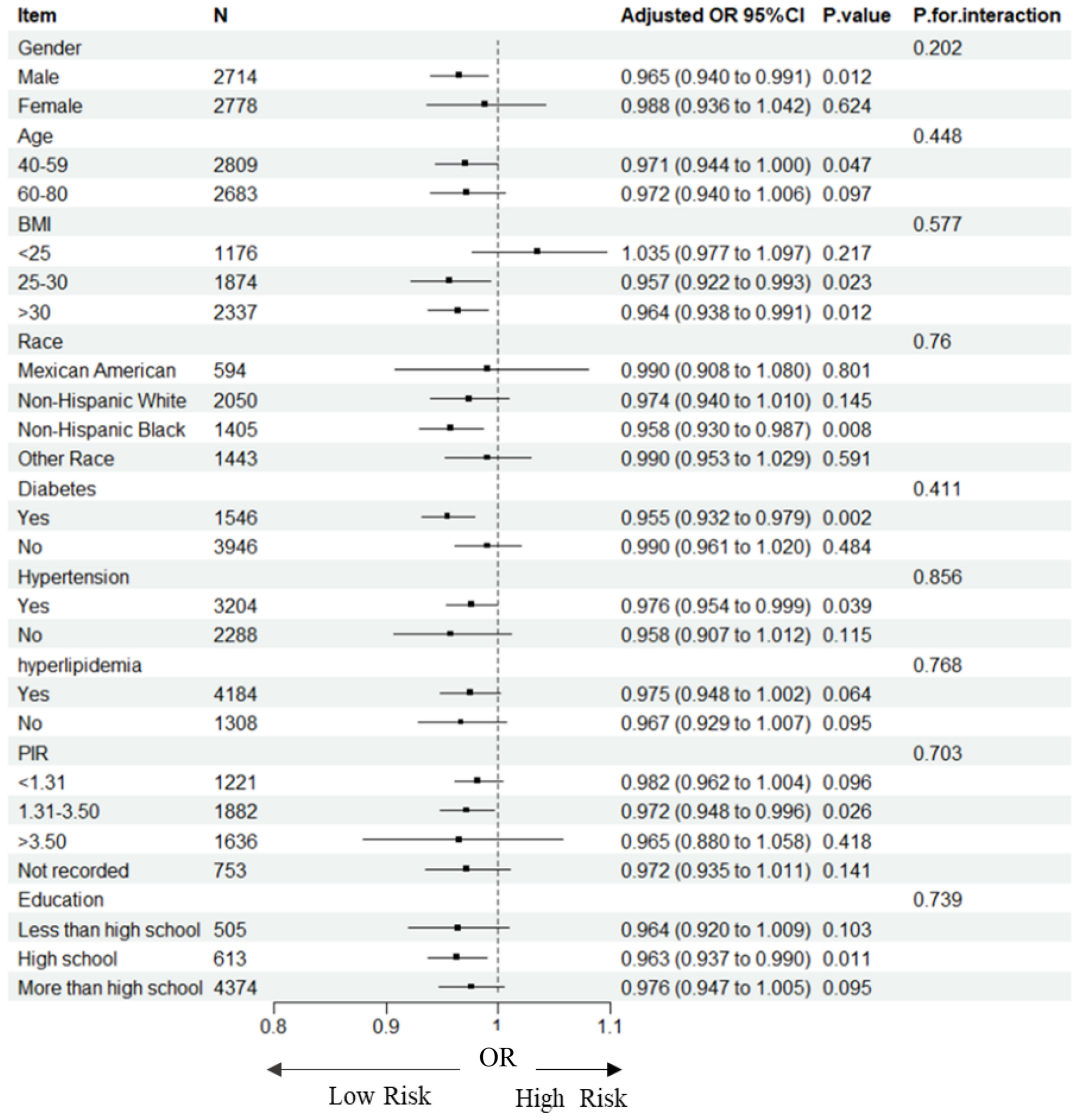
Forest plot for subgroup analysis of association between TSAT and HF. Odds ratios (ORs) were calculated using multivariate Logistic regression models adjusted in Model3. In each case, the stratification variable itself in each subgroup analysis was not included in the adjusted model. The square represents the OR value; the solid line represents 95% confidence interval (CI); the dotted line represents invalid lines.

We investigated the potential relationships between TSAT with HF across various subgroups to further assess the robustness of the association (**Table 3**, **Figure 2**). Significant associations between TSAT and HF were observed in the following subgroups: male sex(OR = 0.965, 95%CI 0.940,0.991), 40-59 age (OR = 0.971, 95%CI 0.944,1.000),25≤BMI ≤ 30(OR = 0.957, 95%CI 0.922,0.993), BMI>30(OR = 0.964, 95%CI 0.938,0.991), non-Hispanic black(OR = 0.958, 95%CI 0.930,0.987), diabetes(OR = 0.955, 95%CI 0.932,0.979), hypertension (OR = 0.976, 95%CI 0.954,0.999), 1.3≤PIR ≤ 3.5(OR = 0.972, 95%CI 0.948,0.996), and high school education level (OR = 0.963, 95%CI 0.937,0.990). Notably, no significant interactions were observed for gender, age, BMI, race, diabetes, hypertension, hyperlipidemia, PIR and education suggesting that the association was not dependent on these variables (all p for interaction > 0.05).

**Table 3:** The results of subgroup analysis between TSAT and HF.

Outcome	Model 1		Model 2		Model 3		P for interaction
	OR (95%CI)	P value	OR (95%CI)	P value	OR (95%CI)	P value	
Gender							0.202
Male	0.948(0.924,0.973)	0.000	0.951(0.926,0.977)	0.001	0.965(0.940,0.991)	0.012	
Female	0.984(0.947,1.021)	0.373	0.977(0.930,1.026)	0.331	0.988(0.936,1.042)	0.624	
Age							0.448
40-59	0.973(0.952,0.995)	0.017	0.958(0.930,0.987)	0.008	0.971(0.944,1.000)	0.047	
60-80	0.966(0.935,0.998)	0.037	0.962(0.931,0.993)	0.019	0.972(0.940,1.006)	0.097	
BMI							0.577
<25	1.022(0.963,1.085)	0.451	1.026(0.957,1.101)	0.447	1.035(0.977,1.097)	0.217	
25-30	0.962(0.934,0.992)	0.015	0.954(0.924,0.985)	0.006	0.957(0.922,0.993)	0.023	
>30	0.970(0.953,0.987)	0.001	0.957(0.932,0.982)	0.002	0.964(0.938,0.991)	0.012	
Race							0.760
Mexican American	0.989(0.927,1.055)	0.719	0.984(0.905,1.070)	0.690	0.990(0.908,1.080)	0.801	
Non- Hispanic White	0.969(0.942,0.996)	0.028	0.962(0.931,0.995)	0.025	0.974(0.940,1.010)	0.145	
Non-Hispanic Black	0.962(0.944,0.981)	0.000	0.952(0.928,0.977)	0.001	0.958(0.930,0.987)	0.008	
Other Race	0.980(0.939,1.023)	0.346	0.972(0.926,1.020)	0.233	0.990(0.953,1.029)	0.591	
Diabetes							0.411
Yes	0.964(0.944,0.985)	0.002	0.955(0.933,0.977)	0.000	0.955(0.932,0.979)	0.002	
No	0.986(0.963,1.010)	0.250	0.981(0.952,1.012)	0.217	0.990(0.961,1.020)	0.484	
Hypertension							0.856
Yes	0.975(0.956,0.994)	0.014	0.968(0.947,0.990)	0.007	0.976(0.954,0.999)	0.039	
No	0.972(0.934,1.012)	0.163	0.958(0.910,1.009)	0.102	0.958(0.907,1.012)	0.115	
Hyperlipidemia							0.768
Yes	0.968(0.947,0.990)	0.007	0.961(0.935,0.987)	0.006	0.975(0.948,1.002)	0.064	
No	0.976(0.947,1.005)	0.099	0.965(0.926,1.005)	0.083	0.967(0.929,1.007)	0.095	
PIR							0.703
<1.31	0.980(0.958,1.002)	0.069	0.974(0.950,0.998)	0.038	0.982(0.962,1.004)	0.096	
1.31-3.50	0.972(0.948,0.996)	0.023	0.966(0.939,0.993)	0.017	0.972(0.948,0.996)	0.026	
>3.50	0.963(0.905,1.025)	0.226	0.949(0.870,1.036)	0.230	0.965(0.880,1.058)	0.418	
Not recorded	0.980(0.943,1.018)	0.285	0.963(0.925,1.003)	0.066	0.972(0.935,1.011)	0.141	
Education							0.739
Less than high school	0.965(0.922,1.010)	0.119	0.959(0.917,1.003)	0.068	0.964(0.920,1.009)	0.103	
High school	0.963(0.948,0.978)	<.001	0.955(0.936,0.974)	<.001	0.963(0.937,0.990)	0.011	
More than high school	0.973(0.951,0.995)	0.019	0.964(0.936,0.992)	0.015	0.976(0.947,1.005)	0.095	

Model 1: no covariates were adjusted. Model 2: Age, gender and race were adjusted. Model 3: All demographic information were adjusted.

### 3.4 Identification of non-linear relationship

**Figure 3:**
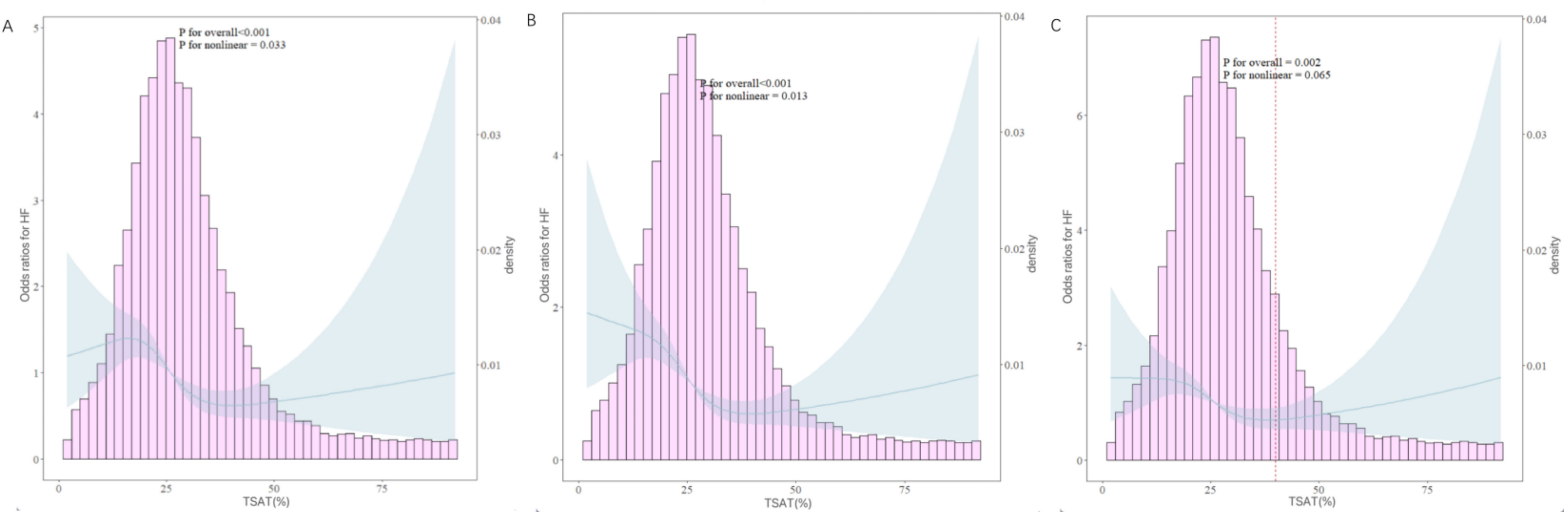
RCS curves between the TSAT and HF. (**A**: adjusted in Model 1; **B**: adjusted in Model 2; **C**: adjusted in Model 3). The solid blue lines represented the ORs of HF, the blue region indicated corresponding 95% Cis, the pink histogram showed the distribution of TSAT. The left Y-axis represents the OR of HF, the right Y-axis represents the frequency distribution of TSAT, the X-axis is the TSAT value. The picture show both the frequency distribution of data and the fitting curve of RCS model. The P-value for overall association <0.05 manifested a significant association, whatever the shape of the dose-response curve was. The P-value for non-linear association <0.05 indicated a nonmonotonic dose-response curve.

We further explored the dose–response relationship between TSAT and HF using RCS plots, and the results are shown in **Figure 3**. We built three RCS plots according to Model 1, Model 2 and Model 3. **Figures 3A**, **B** showed a nonlinear dose–response relationship between TSAT and HF (P for nonlinearity = 0.033, P for nonlinearity = 0.013), while **Figure 3C** showed a linear dose–response relationship between TSAT and HF (P for nonlinearity = 0.065). A threshold effect analysis of TSAT on HF was further performed by the two-piecewise linear regression. As shown in **Table 4**, the inflection point was 40. Each unit increase of TSAT was associated with a 3.0% decrease of the risk of HF below 40, and the relationship was not statistically significant above 40.

**Table 4:** Threshold effect analysis of TSAT and HF.

TSAT(%)	Adjust OR (95%CI) P value
Mode 1 Fitting model by standerd linear regression	0.980(0.967,0.992) 0.002
Mode 2 Fitting model by two-piecewise linear regression	
Inflection point	40
<40	0.970(0.955,0.985) <0.001
>40	1.022(0.984,1.054) 0.205
P for likelihood ratio test	0.024

## 4 Discussion

To illuminate the correlation between TSAT and HF incidence, we conducted a cross-sectional analysis involving 5,492 individuals from NHANES 2017-2020.03. Our study revealed an inverse association between TSAT and HF. Specifically, for each 1% increase in TSAT, the risk of HF decreased by 2.6% after adjusting for covariates. This negative and significant association persisted in approximately half of the subgroups examined. To assess the linearity of this relationship, we employed restricted cubic spline regression and identified a negative linear relationship between TSAT and HF. Threshold effect analysis suggested that the inflection point was 40.

In addition, our study found that compared with non-HF patients, patients with HF had lower ALT, albumin, serum iron, hemoglobin and higher CRP. This is consistent with previous studies that low ALT, albumin, hemoglobin and serum iron are associated with adverse outcomes in patients with HF (22–25). However, no significant differences were found between the two groups in terms of dietary intake and sedentary time, which we think may be due to the fact that these data were obtained through recall. More RCT studies or cohort studies are needed to verify the effects of dietary intake and sedentary time on patients with HF.

TSAT serves as a measure of the percentage of transferrin saturated with iron, reflecting the availability of iron for erythropoiesis and myocardial tissue (26). Although existing research on the relationship between TSAT and HF incidence is still relatively rare, the use of intravenous iron supplementation to treat HF has become a hot topic of research today (27–29). While ID is known for its associations with clinical consequences related to erythropoiesis, chronic ID by itself, independent of anemia, poses detrimental effects on oxidative metabolism, cellular energetics, and immune mechanisms, consequently leading to structural and functional changes in the myocardium. This includes reductions in oxygen storage within myoglobin and diminished tissue oxidative capacity, ultimately resulting in mitochondrial and left ventricular (LV) dysfunction (30). Consequently, it’s not surprising that ID correlates with LV hypertrophy, dilatation, compromised hemodynamics and symptomatic HF (31).

The underlying mechanism linking TSAT and HF remains unclear. Some animal studies have demonstrated that ID induces diastolic dysfunction and HF, characterized by pulmonary congestion, cardiac fibrosis, and increased cardiac inflammation (32). For instance, Toblli et al. established an ID model in rats by feeding them a low-iron diet, leading to significantly reduced serum iron and TSAT levels compared to rats fed normal diets. Echocardiography and tissue Doppler evaluations revealed significantly increased left ventricular end-diastolic diameter, left ventricular end-systolic diameter, left ventricular posterior wall thickness, and decreased S and E waves velocities (33). Similarly, Chung et al. found that iron-deficient animals exhibited reduced ventricular ejection fraction measured by magnetic resonance imaging, and disturbances in Ca 2+ signaling, a pathway linked to the contractile deficit in failing hearts. This was attributed to downregulated RyR2 channels, decreased sarcoplasmic reticulum release, dephosphorylation of phosphoprotein, and suppressed SERCA pump activity (34). ID has also been implicated in increasing oxidative/nitrosative stress in the heart, leading to aberrant mitochondrial and irregular sarcomere organization, and cytochrome c release. This oxidative/nitrosative stress arises from increased mitochondrial and enzymatic superoxide (O2• −) production, enhanced inducible nitric oxide synthase expression, and subsequent peroxynitrite (ONOO−) formation (29). Clinical studies have further supported these findings. For example, Zhang et al. reported lower concentrations of cardiac iron in heart tissue of patients with HF compared to those without HF. This depletion of cardiac iron is believed to be associated with inhibited respiratory chain and Krebs cycle enzyme activity, depletion of iron storage, and an increase in malondialdehyde concentration (35).

In our subgroup analysis, we found that the association between TSAT and HF was more pronounced in participants with high school education compared to other education. The possible reason is that people with high school education have stronger self-management skills than people with less than high school education, allowing them to more effectively adjust their lifestyle, follow medical advice or take other preventive measures to reduce the risk of HF (36); while people with high school education have lower psychological stress than those with more than high school education, which reduce the occurrence of HF (37). This result is also consistent with the PIR in this study. However, the underlying mechanisms for this education-related effect of TSAT on HF are not yet well understood and more research is needed.

However, our study had several limitations: (1) the specific types and NYHA classification of HF were unknown, and the data of NT-pro brain natriuretic peptide (BNP) or BNP was not found. (2) The participants included in our study were middle-aged and older individuals from the United States, and therefore, the generalizability of our findings to other age groups and national populations requires further investigation. (3) the factors influencing both TSAT and HF are multifaceted, and despite adjusting for relevant covariates in our models based on existing literature, the possibility of bias from unaccounted potential covariates cannot be ruled out. (4) The cross-sectional nature of our study limits our ability to establish causality. Further prospective studies and randomized controlled trials are needed to confirm the link between TSAT and HF.

## 5 Conclusions

Our study found a linear negative association between TSAT and the presence of HF among middle-aged and older adults, after being adjusted for potential confounders. This finding highlights the importance of improve TSAT in HF patients to prevent disease progression. In the future, additional randomized controlled trials or cohort studies are urgently needed to validate our conclusions and further explore the underlying mechanisms of this association.

## Data Availability

The original contributions presented in the study are included in the article/supplementary material. Further inquiries can be directed to the corresponding author.
